# Supplementary material for: Global metabolic rewiring for improved CO2 fixation and chemical production in cyanobacteria
Source: Nat Commun. 2017 Mar 13;8:14724. doi: 10.1038/ncomms14724 (PMC5355792; doi:10.1038/ncomms14724)
Supplement: Supplementary Information — Supplementary Figures, Supplementary Tables, Supplementary Notes, Supplementary References. [file ncomms14724-s1.pdf]

Supplementary Table 1 Oligonucleotides used in this study

Name	Sequence (5'-3')
ALC11	GAAAAGTGCCACCTGACGTCCCAAGTTTTCATCTCAGTTT
ALC12	AAACTGAGATGAAAACTTGGGACGTCAGGTGGCACTTTTC
ALC13	CTTCCCTCAACTCGATCCTGGGGCGTGGCGATCGCAAAAG
ALC14	CTTTTGCGATCGCCACGCCCCAGGATCGAGTTGAGGGAAG
ALC15	GTTCTGCTGGCTAGGAACGCGATCCCTGCAACCCAGCTCA
ALC16	TGAGCTGGGTTGCAGGGATCGCGTTCCTAGCCAGCAGAAC
ALC17	GACCAAGAAACCTACATCGCTCTAGAGGCATCAAATAAAA
ALC18	TTTTATTTGATGCCTCTAGAGCGATGTAGGTTTCTTGGTC
MC177	CAAACAGAGAGGCATTGCCGGTTTGAGCGA
MC178	GCCGCCGAGTTGGCTATCGCTTGGTCA
MC181	CGAGTGGCTGTACAACGGTGGTCCCTAC
IM89	GTTGGGTAGCAGACAATGCGGGGGATCTGG
MK139	CGACTGCACGGTGCACCAAT
MK140	CTGTTTCCTGTGTGAAATTGTTAT
MK141	ATTGGTGCACCGTGCAGTCGCTCGAGTCTCATCCCTAAC
MK142	CAATTTCACACAGGAAACAGGAATTCATTAAAGAGGAGAAAGG
MK237	TGCAGGGACGGACGGTCTTTATCATTGCTC
MK238	ATCGCTGATTCTCGATTGCCTGCGTTATTG
MK372	TTGCACCAGGATCCCGCTCGCGACTGCACGGTGCACCAAT
MK373	TTGATGCCTCTAGAACGCGTGAGAGCGTTCACCGACAAAC
MK374	GTTTGTCGGTGAACGCTCTCACGCGTTCTAGAGGCATCAA
MK377	TGCACCGTGCAGTCGCGAGCGGGATCCTGGTGCAA
MK380	CAGGAAACAGGAATTCATTAGTCGACGAGGAATCACCATG
MK381	AGGTCGACTCTAGAGGATCTCTACAGGATTACGACGGCTTTA
MK382	AAGCCGTCGTAATCCTGTAGAGATCCTCTAGAGTCGACCT
MK383	CATGGTGATTCCTCGTCGACTAATGAATTCCTGTTTCCTG
MK388	ACATTGAGCAACTGACTGAAATGCCTCAAA
MK389	GGACAACTTCTTCGCCCCCGTTTTCACCAT
MK442	TTCCCCGAAAAGTGCCACCTCGATCGGTGGTGATGGTTCA
MK443	TGCTCTCGGTCTTTTGCGATAGACAAGCTGGGAGCGACCT
MK444	AGGTCGCTCCCAGCTTGTCTATCGCAAAAGACCGAGAGCA
MK445	TTCCCCACTCGCATGAGGATGCGTTCCTAGCCAGCAGAAC
MK446	TGCTGGCTAGGAACGCATCCTCATGCGAGTGGGGAAAATCA
MK447	TTTTATTTGATGCCTCTAGGTTGAACTACGCGCCAACAAC
MK448	GTTGTTGGCGCGTAGTTCAACCTAGAGGCATCAAATAAAA
MK449	GAACCATCACCACCGATCGAGGTGGCACTTTTCGGGGAAAT
MK489	ATTGTGAGCGGATAACAATTAGCATTTTTGCTGGAGGAAAACC
MK490	GCAGTTCCCTACTCTCGCTTTTAGTAACGGGTTTGGTTGG
MK491	CCAACCAAACCCGTTACTAAAAGCGAGAGTAGGGAACTGC
MK492	TTCCTCCAGCAAAAATGCTAATTGTTATCCGCTCACAATTC
MK496	GACAGCGTTTGCTAGTGTGACGTACGCTCGAACCCCAGAGTC
MK497	TGAGCGTTGATTGAGGTGAGAGAGCGTTCACCGACAAACA

MK498	TGTTTGTCGGTGAACGCTCTCTCACCTCAATCAACGCTCA
MK499	CTCTGGGGTTCGAGCGTACGTCACACTAGCAAACGCTGTC
MK504	ATTGTGAGCGGATAACAATTAACCATATTGGAGGGCATCA
MK505	GCAGTTCCCTACTCTCGCTTTTAATCGTGAGCGCCTATTT
MK506	AAATAGGCGCTCACGATTAAAAGCGAGAGTAGGGAACTGC
MK507	TGATGCCCTCCAATATGGTTAATTGTTATCCGCTCACAAT
MK570	GATTGCTACAGCACCAGTGCAGCGCCTTTTAGCGGGCGAT
MK571	ACAGGAGAGTATCGTCAGAAGCAGGAGCGTGATCCCCGCTTCG
MK572	AGCGGGGATCACGCTCCTGCTTCTGACGATACTCTCCTGT
MK573	ACCTTAGAGACTGCTGTCGAGCGTTCCTAGCCAGCAGAAC
MK574	GTTCTGCTGGCTAGGAACGCTCGACAGCAGTCTCTAAGGT
MK575	TTTTATTTGATGCCTCTAGGATCGCAGCCTCGAGCAGATT
MK576	AATCTGCTCGAGGCTGCGATCCTAGAGGCATCAAATAAAA
MK577	ATCGCCCGCTAAAAGGCGCTGCACTGGTGCTGTAGCAATC
MK588	GACTGGAAAGCGGGCAGTGAAGCTCTCGGGTAACATCAAG
MK589	CTTGATGTTACCCGAGAGCTTCACTGCCCGCTTTCCAGTC
MK662	TTTGAATGTATTTAGAAAAAGGCTGCCAGCCCGAAACAGC
MK663	AGTAACAACTTATATCGTATCCCTGCTCGTCACGCTTTCA
MK664	TGAAAGCGTGACGAGCAGGGATACGATATAAGTTGTTACT
MK665	CATCTTCCTGCTCCAGAAGCCTCGCCCAATGGACAAGGGA
MK666	TCCCTTGTCCATTGGGCGAGGCTTCTGGAGCAGGAAGATG
MK667	ACTCAGGAGAGCGTTCACCGATGGATCTGACCAACATGAT
MK668	ATCATGTTGGTCAGATCCATCGGTGAACGCTCTCCTGAGT
MK669	GCTGTTTCGGGCTGGCAGCCTTTTTCTAAATACATTCAAA
MK691	TTGTGAGCGGATAACAATTATTATCTCGTCACTGTCTCGAGG
MK694	AGACAGTGACGAGATAATAATTGTTATCCGCTCACAATTC
MK695	GTCAACCTCTCTCGTCTGACCTCTTGATCTGCGGA
MK696	AGACCTTTGAGTCGATGTTTGGCGTGCCTA
MK777	GAAAAGTGCCACCTGACGTCAGCGATAGAGATGGAGGAGT
MK778	CTTTTGCGATCGCCACGCCCAGGAAAATTCCTGCTAGGAC
MK779	GTCCTAGCAGGAATTTTCCTGGGCGTGGCGATCGCAAAAG
MK780	TCGCCTTGCCAGTCCTGATTGCGTTCCTAGCCAGCAGAAC
MK781	GTTCTGCTGGCTAGGAACGCAATCAGGACTGGCAAGGCGA
MK782	TTTTATTTGATGCCTCTAGAACTACCGTGAGATCGGTCAG
MK783	CTGACCGATCTCACGGTAGTTCTAGAGGCATCAAATAAAA
MK784	ACTCCTCCATCTCTATCGCTGACGTCAGGTGGCACTTTTC
MK793	AAAAGTGCCACCTGACGTCTTCGCGGGCTGCTCAGCTCT
MK794	CTTTTGCGATCGCCACGCCCTTTGACGATCGCTCCAAAAT
MK795	ATTTTGGAGCGATCGTCAAAGGGCGTGGCGATCGCAAAAG
MK796	ACTGGATGAGCGGACATCAAGCGTTCCTAGCCAGCAGAAC
MK797	GTTCTGCTGGCTAGGAACGCTTGATGTCCGCTCATCCAGT
MK798	TTTTATTTGATGCCTCTAGATTCCGGATTTAGCCTTGCTT
MK799	AAGCAAGGCTAAATCCGGAATCTAGAGGCATCAAATAAAA
MK800	AGAGCTGAGCAGCCCGCGAAGACGTCAGGTGGCACTTTTC
MK813	AAATAGGCGCTCACGATTAAACCGGGTTAGTTAACTTAAG

MK814	CTTAAGTTAACTAACCCGGTTTAATCGTGAGCGCCTATTT
MK843	TCCTCCAGCAAAAATGCTCTAGACGCTAGCGGCGACGGGT
MK844	CCGTCGCCGCTAGCGTCTAGAGCATTTTTGCTGGAGGAAAACC
MK944	TTACGTGCCACCGGTATCCTTGGCGGCAAGAAAGC
MK945	TTTTATTTGATGCCTCTAGGTAAGCGGGCCACGGCAGCGAA
MK946	TCGCTGCCGTGGCCCGCTTACCTAGAGGCATCAAATAAAA
MK947	TCCAAGTCCCAAAGCGATCGTTTATTGGTGAGAATCCAAG
MK948	CTTGGATTCTCACCAATAAACGATCGCTTTGGGACTTGGA
MK949	CTCGGTTGCCGCCGGGCGTTTGAAGTTTGCATTGTTTTTA
MK950	TAAAAACAATGCAAACTTCAAACGCCCGGCGGCAACCGAG
MK951	CTTTCTTGCCGCCAAGGATACCGGTGGCACGTAAGAGGTT
MK978	AAATAGGCGCTCACGATTAACTCAAAATCAGAAGAGTATT
MK979	GCAGTTCCCTACTCTCGCTTTTAACCGCGCCACGCTTTAT
MK980	ATAAAGCGTGGCGCGGTTAAAAGCGAGAGTAGGGAACTGC
MK981	AATACTCTTCTGATTTTGAGTTAATCGTGAGCGCCTATTT
MK1000	CCTGGAATGAGTTTGAGTAAAGCGCGGTGATCACACCTGA
MK1001	GCAGTTCCCTACTCTCGCTTTTAATCCAGCCATTCGGTAT
MK1002	ATACCGAATGGCTGGATTAAAAGCGAGAGTAGGGAACTGC
MK1003	TCAGGTGTGATCACCGCGCTTTACTCAAACTCATTCCAGG
MK1006	CCTGGAATGAGTTTGAGTAACTGACAACTCAATTTCAGGA
MK1007	GCAGTTCCCTACTCTCGCTTTTAAAAAGTGATACAGGTTG
MK1008	CAACCTGTATCACTTTTTAAAAGCGAGAGTAGGGAACTGC
MK1009	TCCTGAAATTGAGTTGTCAGTTACTCAAACTCATTCCAGGAACG
MK1105	ATCAGCAGGACGCACTGACCTTCTCAAAGGAGAGTTATCA
MK1136	ATTGTGAGCGGATAACAATTTCCAAAGTTCAGAGGTAGTC
MK1137	GCAGTTCCCTACTCTCGCTTTTAATACAGTTTTTTCGCGCAGTC
MK1138	GCGCGAAAAAACTGTATTAAAAGCGAGAGTAGGGAACTGC
MK1139	GACTACCTCTGAACTTTGGAAATTGTTATCCGCTCACAATTC
MK1146	ATTGTGAGCGGATAACAATTTCAGGCGAGAGAAAACTCTG
MK1147	GCAGTTCCCTACTCTCGCTTTTACAGCTTAGCGCCTTCTA
MK1148	TAGAAGGCGCTAAGCTGTAAAAGCGAGAGTAGGGAACTGC
MK1149	CAGAGTTTTCTCTCGCCTGAAATTGTTATCCGCTCACAATTC

Supplementary Table 2 Plasmids used in this study

**Plasmids**		
pAL34	*cp12* knockout vector; kan^R; ColE1	This study
pAL40	NSI targeting vector; $lacI^q$; *P*trc: *galP*; ColE1; $spec^R$	1
pAL321	*cp12* knockout vector; cm^R; ColE1	This study
pAL552	NSIII targeting vector; $lacI^q$; $P_L$lacO$_1$: *sfgfp*; ColE1; $gent^R$	2
pAL792	NSIII targeting vector; $lacI^q$; *P*trc: *gpmM-eno-pykA*; ColE1; kan^R	3
pAL979	NSII targeting vector; $lacI^q$; $P_L$lacO$_1$: *pyk*; ColE1; kan^R	3
pAL991	$P_L$lacO$_1$: *ATF1*; p15A; $spec^R$	4
pAL1040	NSIII targeting vector; $lacI^q$; $P_L$lacO$_1$: *alsD-alsS-adh*; ColE1; $gent^R$	2
pAL1126	NSIII targeting vector; $lacI^q$; *P*trc: *sfgfp*; ColE1; $gent^R$	This study
pAL1136	NSIII targeting vector; $lacI^q$; $P_L$lacO$_1$: *alsD-alsS-adh*; ColE1; $gent^R$	This study
pAL1200	NSII targeting vector; $lacI^q$; *P*trc: *galP*; ColE1; kan^R	This study
pAL1211	NSII targeting vector; $lacI^q$; *P*trc: *rbcLXS*; ColE1; kan^R	This study
pAL1215	*cp12* knockout vector; $lacI^q$; *P*trc: *rbcLXS*; kan^R	This study
pAL1300	*pfk* knockout vector; cm^R	This study
pAL1314	NSI targeting vector; $lacI^q$; *P*trc: *galP*; ColE1; $spec^R$	This study
pAL1357	*pgi* knockout vector; kan^R	This study
pAL1361	*zwf* knockout vector; kan^R	This study
pAL1363	*gnd* knockout vector; kan^R	This study
pAL1364	*eda* knockout vector; kan^R	This study
pAL1397	*cp12* knockout vector; $lacI^q$; *P*trc: *prk-rbcLXS*; kan^R	This study
pAL1448	NSI targeting vector; $lacI^q$; *P*trc: *galP-zwf-edd*; ColE1; $spec^R$	This study
pAL1449	NSI targeting vector; $lacI^q$; *P*trc: *galP-pgi*; ColE1; $spec^R$	This study
pAL1450	NSI targeting vector; $lacI^q$; *P*trc: *galP-zwf-gnd*; ColE1; $spec^R$	This study
pAL1484	NSII targeting vector; $lacI^q$; *P*trc: *pfkA*; ColE1; kan^R	This study
pAL1486	NSII targeting vector; $lacI^q$; *P*trc: *eda*; ColE1; kan^R	This study

Supplementary Table 3 Plasmid construction by SLIC

Plasmid	PCR					
	Primers	Template	Fragment	Primers	Template	Fragment
**pAL1126**	MK139/MK140	pAL40	*P*trc	MK141/MK142	pAL552	backbone
**pAL1133**	MK372/MK373	pAL792	*P*trc: *gpmM-eno-pykA*	MK374/MK377	pAL979	backbone
**pAL1136**	MK380/MK381	pAL1040	*P*trc: *alsD-alsS-adh*	MK382/MK383	pAL1126	backbone
**pAL1200**	MK504/MK505	*E.coli* gDNA	*galP*	MK506/MK507	pAL1133	backbone
**pAL1211**	MK489/MK490	*Synechococcus* sp. PCC 7002 gDNA	*rbcLXS*	MK491/MK492	pAL1133	backbone
**pAL1215**	MK496/MK497	pAL1211	*kan*^{R}; *lacI*^{q}; *P*trc: *rbcLXS*	MK498/MK499	pAL321	backbone
**pAL1300**	MK570/MK571	*S. elongatus* gDNA	5' homology arm	MK576/MK577	pAL321	backbone
	MK572/MK573	pAL321	*cm*^{R}	MK574/MK575	*S. elongatus* gDNA	3' homology arm
**pAL1314**	MK662/MK663	*S. elongatus* gDNA	5' NSI	MK664/MK588	pAL991	*spec*^{R}
	MK666/MK667	*S. elongatus* gDNA	3' NSI	MK668/MK669	pAL321	backbone
	MK589/MK665	pAL1200	*lacI*^{q}; *P*trc: *galP*			
**pAL1357**	MK442/MK443	*S. elongatus* gDNA	5' homology arm	MK444/MK445	pAL34	*kan*^{R}
	MK446/MK447	*S. elongatus* gDNA	3' homology arm	MK448/MK449	pAL34	backbone
**pAL1361**	ALC11/ALC14	*S. elongatus* gDNA	5' homology arm	ALC13/ALC16	pAL34	*kan*^{R}
	ALC15/ACL18	*S. elongatus* gDNA	3' homology arm	ALC12/ALC17	pAL34	backbone
**pAL1363**	MK777/MK778	*S. elongatus* gDNA	5' homology arm	MK779/MK780	pAL34	*kan*^{R}
	MK781/MK782	*S. elongatus* gDNA	3' homology arm	MK783/MK784	pAL34	backbone
**pAL1364**	MK793/MK794	*S. elongatus* gDNA	5' homology arm	MK795/MK796	pAL34	*kan*^{R}
	MK797/MK798	*S. elongatus* gDNA	3' homology arm	MK799/MK800	pAL34	backbone
**pAL1397**	MK691/MK843	*S. elongatus* gDNA	*prk*	MK844/MK497	pAL1215	*rbcLXS*
	MK498/MK694	pAL1215	backbone			

**pAL1448**	MK813/MK1009	*E. coli* gDNA	*zwf*	MK1006/MK1007	*E. coli* gDNA	*edd*
	MK1008/MK814	pAL1314	backbone			
**pAL1449**	MK978/MK979	*E. coli* gDNA	*pgi*	MK980/MK981	pAL1314	backbone
**pAL1450**	MK813/MK1003	*E. coli* gDNA	*zwf*	MK1000/MK1001	*E. coli* gDNA	*gnd*
	MK1002/MK814	pAL1314	backbone			
**pAL1484**	MK1136/MK1137	*E. coli* gDNA	*pfkA*	MK1138/MK1139	pAL1200	backbone
**pAL1486**	MK1146/MK1147	*E. coli* gDNA	*eda*	MK1148/MK1149	pAL1200	backbone

Supplementary Table 4 Activities of ZWF, GND, PGI, PRK and RuBisCO

Enzymes	Activity (nmol min^{-1} mg^{-1})[a]	
	**Strain 3** (control)[b]	Engineered Strains
ZWF	38 ± 7.3	549 ± 20[c]
GND	21 ± 1.0	74 ± 5.7[c]
PGI	not detectable	125 ± 10[d]
PRK	249 ± 8.5	335 ± 13[e]
RuBisCO	26 ± 1.5	26 ± 1.2[e]

[a] Errors expressed as standard deviation (n=3).

[b] Same as wild type, but with *P*trc: *galP* (NSI) and *P*trc: *alsD-alsS-adh* (NSIII) (**Table 1**).

[c] Same as **Strain 3**, but with *P*trc: *galP-zwf-gnd* (NSI) (**Table 1**).

[d] Same as **Strain 3**, but with *P*trc: *galP-pgi* (NSI) (**Table 1**).

[e] Same as **Strain 3**, but with *P*trc: *prk-rbcLXS* (*cp12*) (**Table 1**)

Abbreviations of enzyme names are as follows: ZWF, glucose-6-phosphate dehydrogenase; GND, 6-phosphogluconate dehydrogenase; PGI, phosphoglucose isomerase; PRK, phosphoribulokinase; RuBisCO, ribulose-1,5-bisphosphate carboxylase/oxygenase.

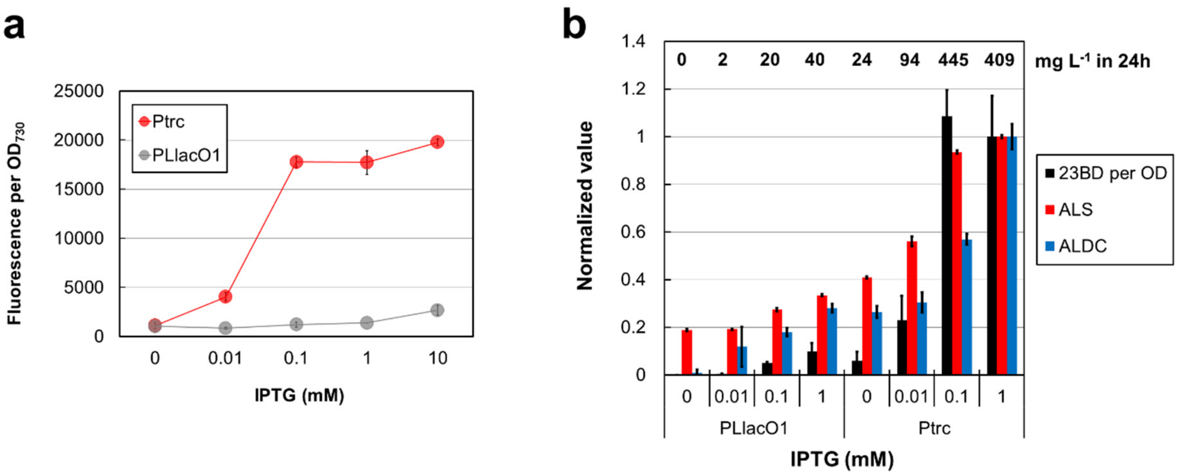

Supplementary Figure 1 Promoter characterization in *S. elongatus*
(**a**) GFP fluorescence assay where **Strains 16** (*P*$_L$lacO$_1$: *sfgfp*) (grey) and **17** (*P*trc: *sfgfp*) (red) (**Table 1**) were used to compare expression between promoters[5, 6]. Cells were diluted to an OD_{730} of 0.1 and allowed to grow for 24 h. Various concentrations of IPTG were added and cultures were allowed to grow for an additional 24 h in continuous light. (**b**) Normalized 23BD production and enzyme activities for each gene in the 23BD production pathway under the control of either *P*$_L$lacO$_1$ (**Strain 2**) or *P*trc (**Strain 1**). Cells were grown in 25 mL of BG11 media containing 50 mM $NaHCO_3$ for 24 h after induction with the specified concentration of IPTG (0, 0.01, 0.1 and 1.0 mM). Values are normalized to those induced with 1.0 mM IPTG in each strain. Bars represent 23BD production (black), and ALS (red) and ALDC (blue) activities. Error bars indicate standard deviation (n=3 biological replicates). The number in bold type above each bar represents actual 23BD production titer of each culture.

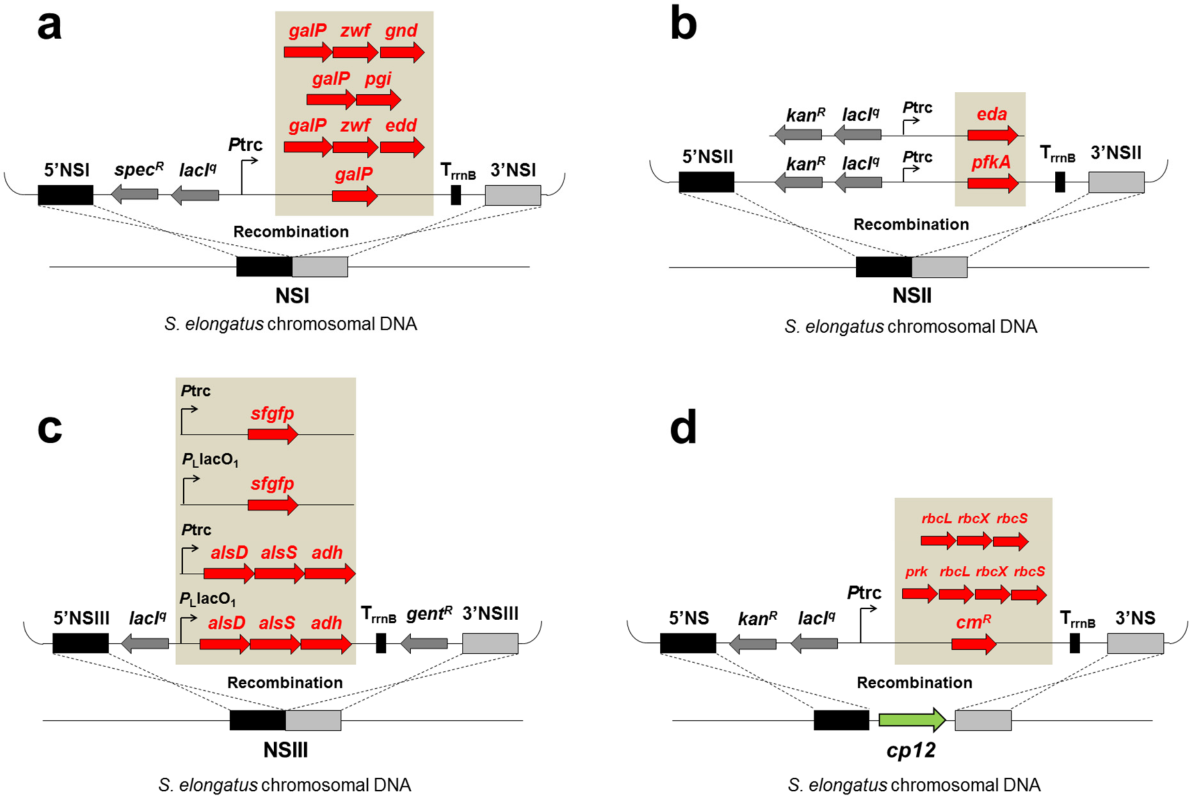

Supplementary Figure 2 Schematic of genome engineering in *S. elongatus*
Schematic of gene integration into (**a**) NSI[7], (**b**) NSII[8], (**c**) NSIII[1], and (**d**) *cp12* (ref. 9). The following plasmids were used for transformation (**Table S2**). (**a**) pAL40 (*galP*), pAL1448 (*galP-zwf-edd*), pAL1449 (*galP-pgi*) and pAL1450 (*galP-zwf-gnd*) were used for modification of NSI. (b) pAL1484 (*pfkA*) and pAL1486 (*eda*) were used for modification of NSII. (c) pAL1040 ($P_L$lacO$_1$: *alsD-alsS-adh*), pAL1136 (*P*trc: *alsD-alsS-adh*), pAL552 ($P_L$lacO$_1$: *sfgfp*) and pAL1126 (*P*trc: *sfgfp*) were used for modification of NSIII. (d) pAL321 (*cp12*:: cm^R), pAL1397 (*cp12*:: *prk-rbcLXS*) and pAL1215 (*P*trc: *rbcLXS*) were used for deletion of *cp12* gene.

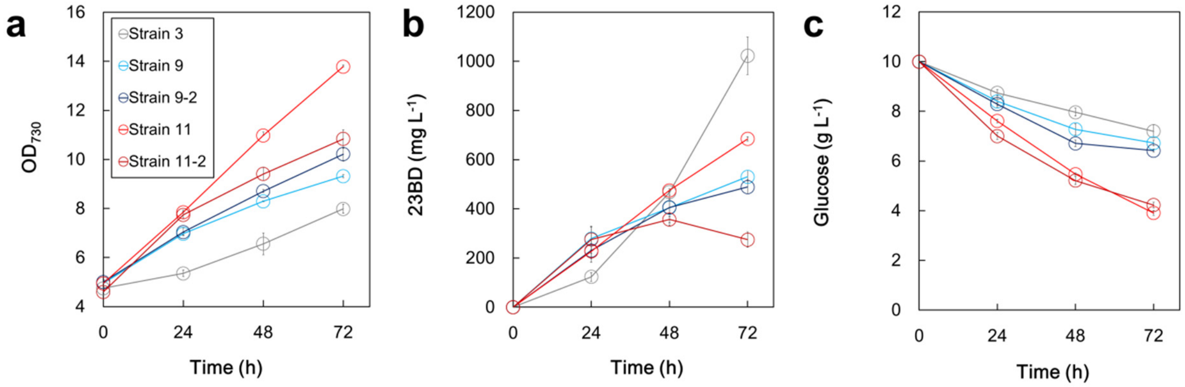

Supplementary Figure 3 Effect of expression of additional pathway genes downstream in the ED pathway and the EMP pathway.

The ED pathway gene, *eda*, and the EMP pathway gene, *pfkA*, are expressed in **Strains 9** (*galP-zwf-edd*) and **10** (*galP-pgi*), respectively, resulting in **Strains 9-2** and **10-2** (**Table 1**). These strains were cultured in 10 mL of BG11 media containing 10 g L^{-1} glucose and 20 mM $NaHCO_3$ under continuous light for 72 h. IPTG (0.1 mM) was added at 0 h. Cell growth (**a**), 23BD concentration (**b**), and glucose consumption (**c**) profiles of **Strain 3** (*galP*, grey), **Strain 9** (*galP-zwf-edd*, light blue), **Strain 9-2** (**9** + *eda*, dark blue), **Strain 10** (*gaP-pgi*, light red) and **Strain 10-2** (**10** + *pfkA*, dark red). N = 3 biological replicates and error bars represent standard deviation.

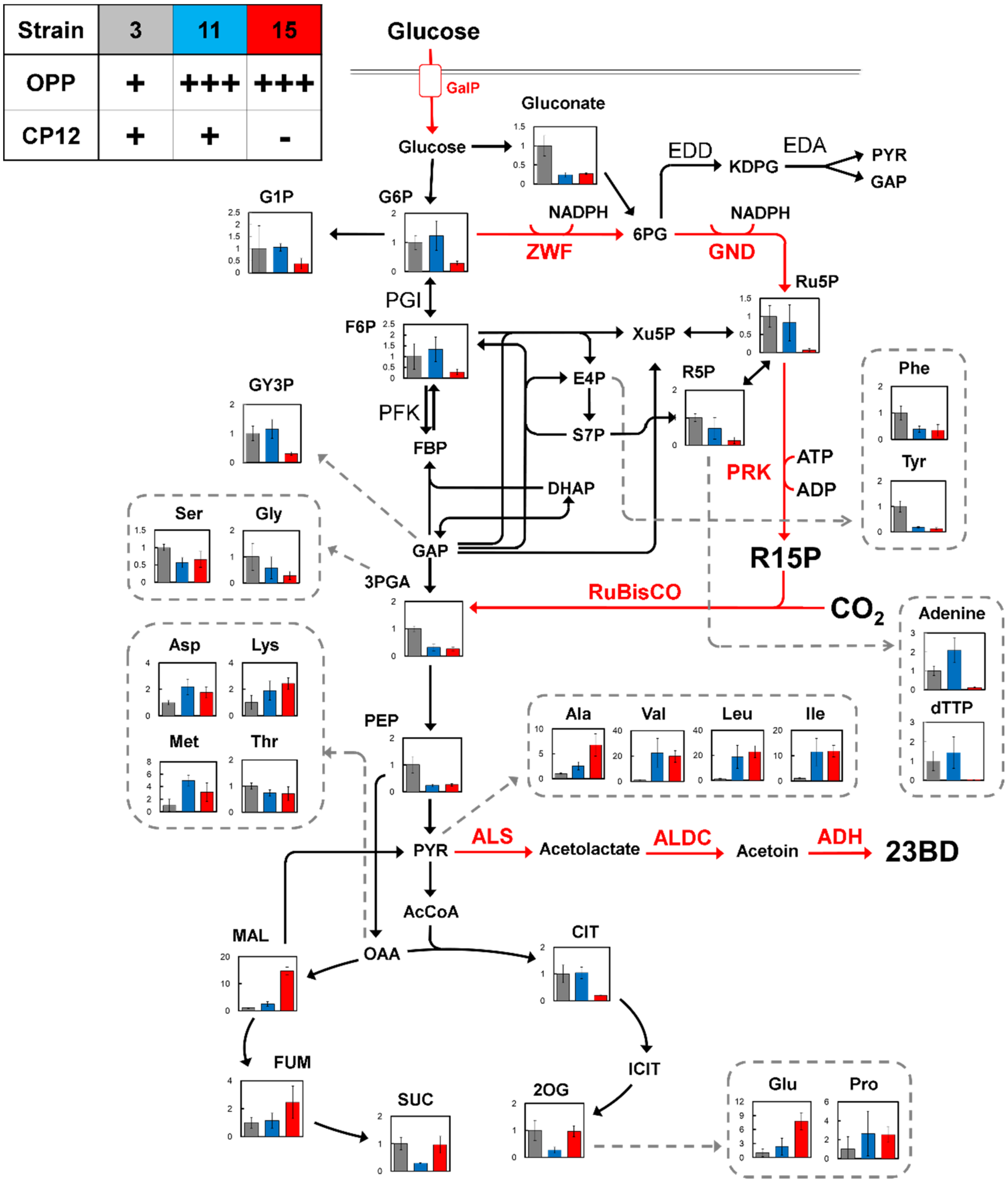

Supplementary Figure 4 Metabolomics data of Strains 3, 11 and 15 grown with glucose in continuous light conditions

Strains 3 (**1** + *galP*), **11** (**1** + *galP-zwf-gnd*) and **15** (**11** + Δ*cp12*:: *prk-rbcLXS*) were cultured in 10 mL of BG11 media containing 10 g L^{-1} glucose and 20 mM $NaHCO_3$ under continuous light conditions for 48 h. IPTG (0.1 mM) was added at 0 h. Intracellular metabolite concentrations of **Strains 3** (grey), **11** (blue) and **15** (red) were measured where n = 3 biological replicates, and error bars represent standard deviation. Strains used for metabolomics analysis are described in the table at the top left: strains harboring *galP* with ('**+++**') and without ('**+**') overexpression of *zwf* and *gnd* (in the row labeled 'OPP') and *cp12* gene natively expressed ('**+**') and replaced with *prk* and *rbcLXS* ('**-**') (in the row labeled 'CP12'). Abbreviations of metabolites and enzymes are the same as those used in **Fig. 1**.

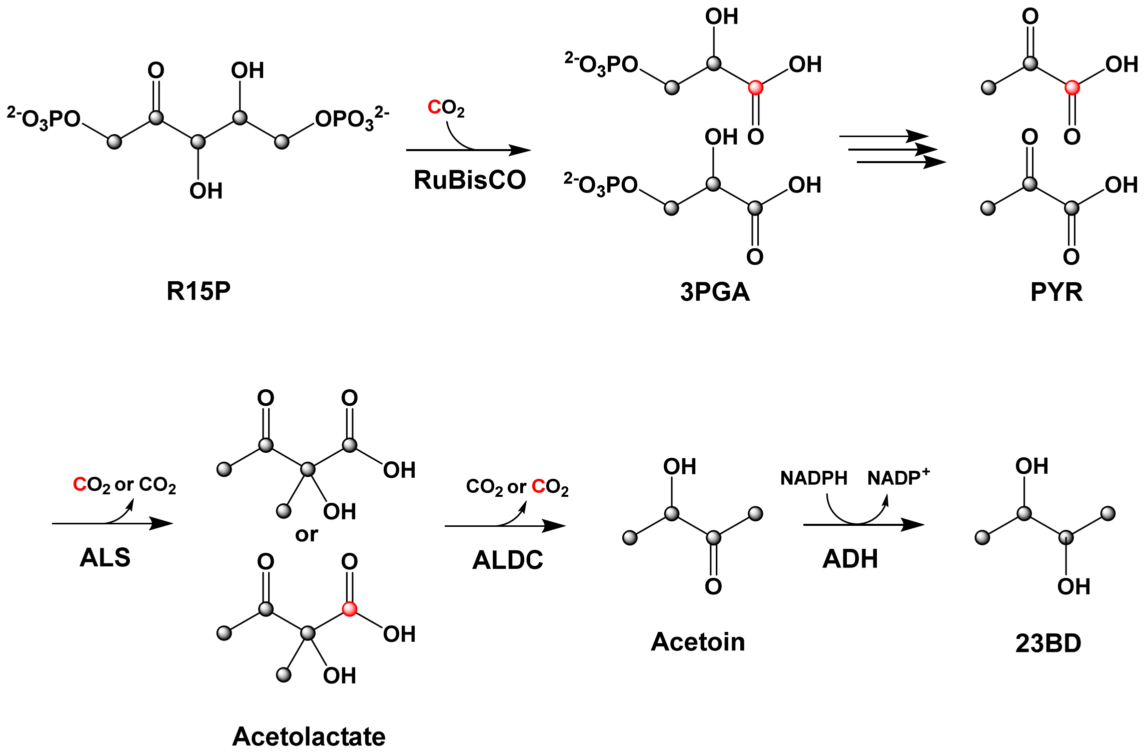

Supplementary Figure 5 Schematic representation of incorporation of $^{13}CO_2$ into 23BD

Because of decarboxylation steps in the 23BD biosynthetic pathway catalyzed by ALS and ALDC, a newly fixed carbon from $^{13}CO_2$ (red) cannot be detected in any of the carbons of 23BD. Metabolite abbreviations are the same as in **Fig. 1**.

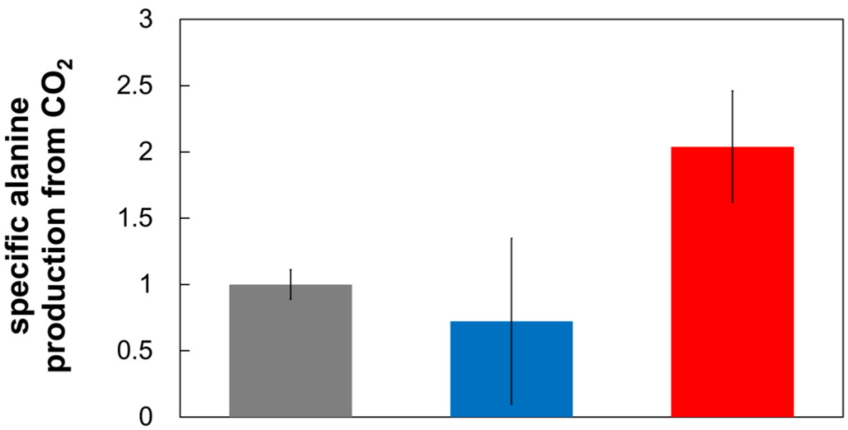

Strain	3	11	15
23BD & *galP*	+	+	+
OPP	+	+++	+++
CP12	+	+	-

Supplementary Figure 6 Specific production of alanine from CO_2 under continuous light conditions

Strains 3 (**1** + *galP*), **11** (**1** + *galP-zwf-gnd*) and **15** (**11** + Δ*cp12*:: *prk-rbcLXS*) were cultured in 10 mL of BG11 media containing 10 g L^{-1} unlabeled glucose and 20 mM ^{13}C-$NaHCO_3$ under continuous light conditions for 48 h and then collected for analysis of the relative amount and ^{13}C labeling ratio of intracellular alanine. IPTG (0.1 mM) was added at 0 h. Specific production of alanine from CO_2 was calculated by multiplying relative amount of intracellular alanine by its ^{13}C labeling ratio in each sample. Strains used in this analysis are described in the table: strains harboring *galP* with (‘**+++**’) and without (‘**+**’) overexpression of *zwf* and *gnd* (in the row labeled ‘OPP’) and *cp12* gene natively expressed (‘**+**’) and replaced with *prk* and *rbcLXS* (‘**-**’) (in the row labeled ‘CP12’). All experiments were performed with biological replicates (n = 3) and error bars represent standard deviation.

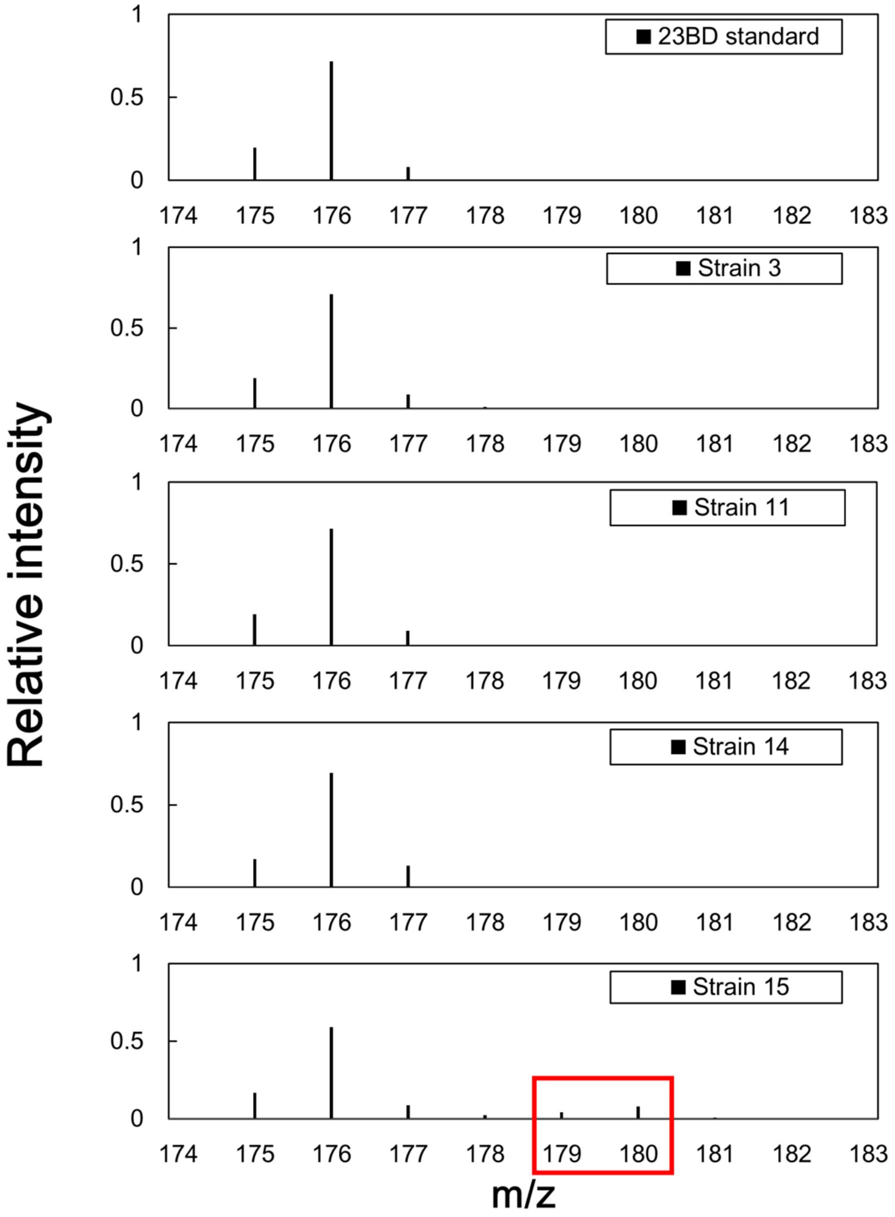

Supplementary Figure 7 Mass spectrum of 23BD produced in continuous dark conditions

Strains 3 (**1** + *galP*), **11** (**1** + *galP-zwf-gnd*), **14** (**3** + Δ*cp12*:: *prk-rbcLXS*) and **15** (**11** + Δ*cp12*:: *prk-rbcLXS*) were cultured in 10 mL of BG11 media containing 10 g L^{-1} unlabeled glucose and 20 mM ^{13}C-$NaHCO_3$ under continuous dark conditions for 24 h. 23BD was derivatized for the analysis by GCMS as described in Methods. IPTG (0.1 mM) was added at 0 h. The MS signals at *m/z* 179 and 180 inside the red square indicate the signals from labeled carbons.

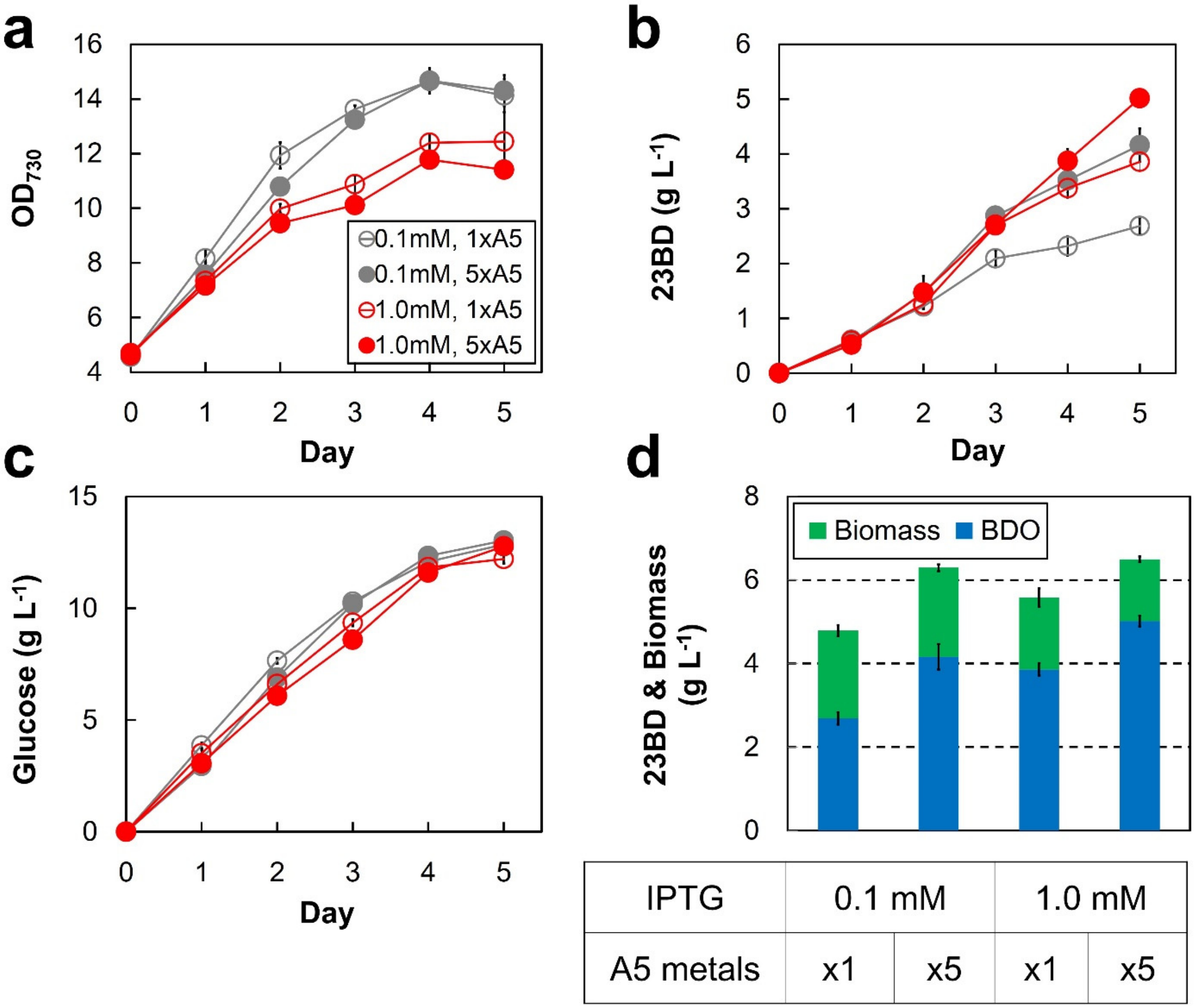

Supplementary Figure 8 Optimization of 23BD production conditions of Strain 15

Strain 15 (**1** + *galP-zwf-gnd* + Δ*cp12*:: *prk-rbcLXS*) was cultured in 10 mL of modified BG11 media containing 15 g L^{-1} glucose and 20 mM $NaHCO_3$ in continuous light conditions. Cell growth (**a**), 23BD concentration (**b**), glucose consumption (**c**) and accumulated 23BD (red bars) and cell biomass (green bars) (**d**) profiles of cells supplemented with 0.1 mM IPTG and 1 x A5 metals (opened grey circles), 0.1 mM IPTG and 5 x A5 metals (closed grey circles), 1.0 mM IPTG and 1 x A5 metals (open red circles) and 1.0 mM IPTG and 5 x A5 metals (closed red circles) where n = 3 biological replicates, error bars represent standard deviation.

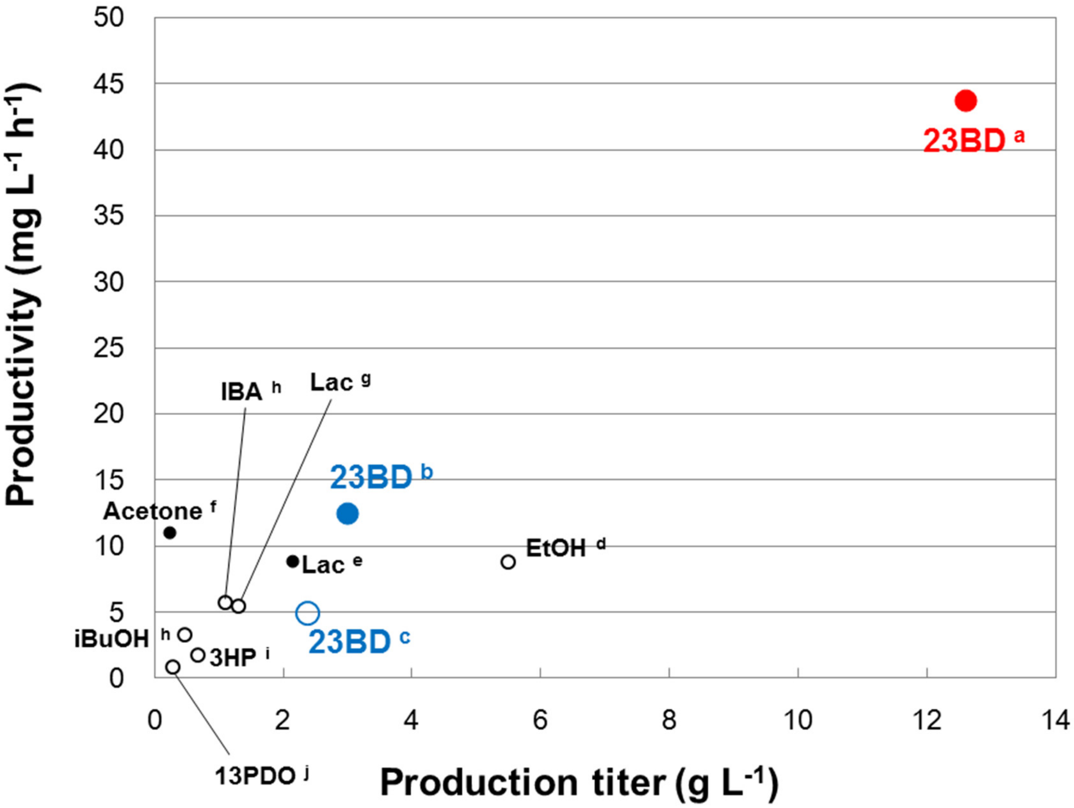

Supplementary Figure 9 Overview of cyanobacterial chemical production
Reported production titers and productivities of chemicals produced by cyanobacteria harboring heterologous biosynthetic pathways. Only relatively high productivities and titers are plotted. Opened circles and closed circles represent chemicals produced photoautotrophically and photomixotrophically, respectively. Besides acetone which was produced with acetate, all other chemicals represented by closed circles were produced with glucose. Productivity was calculated using the presented final titer and the corresponding total production time in the original articles. 23BD production data presented in this study and the previous works are labeled with red and blue, respectively. Abbreviations are as follows: 23BD, 2,3-butanediol; EtOH, ethanol; Lac, D-lactate; iBuOH, isobutanol; IBA, isobutyraldehyde; 3HP, 3-hydroxypropionic acid. [a] this study; [b] McEwen *et al.*[10]; [c] Oliver *et al.*[11]; [d] Gao *et al.*[12]; [e] Verman *et al.*[13]; [f] Chwa *et al.*[14]; [g] Li *et al.*[15]; [h] Atsumi *et al.*[16]; [i] Wang *et al.*[17]; [j] Hirokawa *et al.*[18].

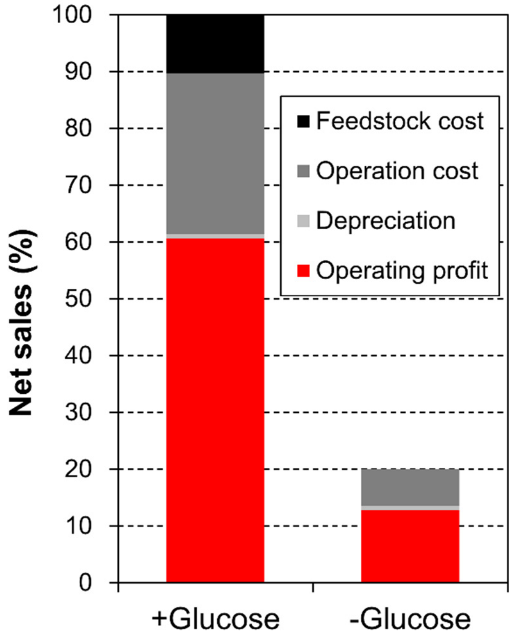

Supplementary Figure 10 Economic feasibility study of photomixotrophic 23BD production

Net sales of 23BD production in photomixtotrophic ('+Glucose') and photoautotrophic ('-Glucose') conditions were calculated assuming each process was operated in diurnal lighting conditions. Operation profit (red) was calculated by subtracting feedstock cost (black), operation cost (dark grey), and depreciation (light grey) from net sales.

Supplementary Note 1

Improvement of the genetic construct for the 23BD biosynthesis pathway

To achieve efficient 23BD production from glucose and CO_2, we first optimized the genetic construct for the 23BD biosynthesis pathway (**Fig. 1c**). In our previous work[11], we demonstrated that the expression of heterologous 23BD biosynthesis pathway genes allowed *Synechococcus elongatus* PCC 7942 (*S. elongatus*) to produce (*R,R*)-23BD from CO_2. Furthermore, we established tight regulation of 23BD production by rearranging the gene order[2] under control of the $P_L$lacO1 promoter[6]. Here, with the goal of improving 23BD production by increasing expression of the pathway genes, we compared 23BD production from strains with the 23BD genes under either $P_L$lacO1 (**Strain 2**) or a stronger promoter, *P*trc[5] (**Strain 1**) (**Supplementary Fig. 1 & 2**). With *P*trc, 23BD production improved by approximately 10-fold, likely due to the strongly enhanced enzymatic activities of the 23BD pathway enzymes (**Supplementary Fig. 1**). For utilization of exogenous glucose, as in our previous work[1], we altered **Strain 1** to express *galP*, which encodes a galactose-proton symporter from *Escherichia coli* (**Strain 3**, **Table 1**). **Strain 3** produced 2.5 g/L of 23BD when glucose was supplied, whereas only 0.4 g/L was produced without glucose (**Fig. 2a & b**). Since this production remarkably exceeded that reported in our previous study with $P_L$lacO1 (~1.0 g/L in 120 h)[10], we used **Strain 3** as a starting point for modifying carbon metabolism.

Supplementary Note 2

Economic analysis of photomixotrophic chemical production

To model the economic feasibility of augmenting a photosynthetic production platform with fixed carbon substrates, principles and assumptions developed by Shiho *et al* for

microalgal oil production[19] were utilized. Operating profit, operation cost, depreciation and feedstock (glucose) cost of an assumed 19-hectare (ha) semi-open pond type plant powered by natural diurnal light (12:12 LD cycle) were calculated. Initially, cells are cultured in plastic membrane tubes placed in the ponds and cell density is maintained at a constant level (1.0 g L^{-1}) by replacing a certain volume of broth with an equal volume of fresh medium. In photomixotrophic production, feedstock cost was additionally estimated assuming $290 per metric ton for DE 95 glucose from corn with a purchase price of $170 per metric ton through the wet-mill process[20]. Furthermore, steam sterilization of production media was calculated for a cost of $2.38 m^{-3} media volume[20]. Removed broth is then subjected further to separation of 23BD. Separation cost was calculated assuming $0.39 kg^{-1} 23BD for the separation via reverse osmosis followed by distillation[21]. To calculate the annual operation cost and total sales, we applied the experimental data (**Fig. 5**) to the model. Therefore, daily specific growth rates of 0.10 day^{-1} and 0.02 day^{-1} in the presence or absence of glucose, respectively, and daily 23BD production per dry cell weight (DCW) of 7.27 g_{23BD}/g_{DCW} for both cases were applied to the model. Based on the amounts of obtained cells and 23BD from 1-year operation, the expense of culture media, mixing, aeration, filtration, steam sterilization and production separation were calculated as described in the original paper. Other parameters, such as room control, property costs, maintenance, general administrative expenses, and labor were the same as described in the original paper. Additionally, 1.07 kg of glucose is required for the photomixotrophic production of 1.00 kg of 23BD and the current market price of 23BD is ~$3 kg^{-1}.

Supplementary references

1. McEwen, J.T., Machado, I.M., Connor, M.R. & Atsumi, S. Engineering *Synechococcus elongatus* PCC 7942 for continuous growth under diurnal conditions. *Appl Environ Microbiol* **79**, 1668-1675 (2013).
2. Nozzi, N.E. & Atsumi, S. Genome engineering of the 2,3-butanediol biosynthetic pathway for tight regulation in cyanobacteria. *ACS Synth Biol* **4**, 1197-1204 (2015).
3. Oliver, J.W. & Atsumi, S. A carbon sink pathway increases carbon productivity in cyanobacteria. *Metab Eng* **29**, 106-112 (2015).
4. Tashiro, Y., Desai, S.H. & Atsumi, S. Two-dimensional isobutyl acetate production pathways to improve carbon yield. *Nat Commun* **6**, 7488 (2015).
5. Brosius, J., Erfle, M. & Storella, J. Spacing of the -10 and -35 regions in the tac promoter. Effect on its *in vivo* activity. *J Biol Chem* **260**, 3539-3541 (1985).
6. Lutz, R. & Bujard, H. Independent and tight regulation of transcriptional units in *Escherichia coli* via the LacR/O, the TetR/O and AraC/I1-I2 regulatory elements. *Nucleic Acids Res* **25**, 1203-1210 (1997).

7. Bustos, S.A. & Golden, S.S. Light-regulated expression of the *psbD* gene family in *Synechococcus* sp. strain PCC 7942: evidence for the role of duplicated *psbD* genes in cyanobacteria. *Mol Gen Genet* **232**, 221-230 (1992).

8. Anderson, S.L. & McIntosh, L. Light-activated heterotrophic growth of the cyanobacterium *Synechocystis* sp. strain PCC 6803: a blue-light-requiring process. *J Bacteriol* **173**, 2761-2767 (1991).

9. Tamoi, M., Miyazaki, T., Fukamizo, T. & Shigeoka, S. The Calvin cycle in cyanobacteria is regulated by CP12 via the NAD(H)/NADP(H) ratio under light/dark conditions. *Plant J* **42**, 504-513 (2005).

10. McEwen, J.T., Kanno, M. & Atsumi, S. 2,3 Butanediol production in an obligate photoautotrophic cyanobacterium in dark conditions via diverse sugar consumption. *Metab Eng* **36**, 28-36 (2016).

11. Oliver, J.W., Machado, I.M., Yoneda, H. & Atsumi, S. Cyanobacterial conversion of carbon dioxide to 2,3-butanediol. *Proc Natl Acad Sci U S A* **110**, 1249-1254 (2013).

12. Gao, Z., Zhao, H., Li, Z., Tan, X. & Lu, X. Photosynthetic production of ethanol from carbon dioxide in genetically engineered cyanobacteria. *Energy Environ Sci* **5**, 9857-9865 (2012).

13. Varman, A.M., Yu, Y., You, L. & Tang, Y.J. Photoautotrophic production of D-lactic acid in an engineered cyanobacterium. *Microb Cell Fact* **12**, 117 (2013).

14. Chwa, J.W., Kim, W.J., Sim, S.J., Um, Y. & Woo, H.M. Engineering of a modular and synthetic phosphoketolase pathway for photosynthetic production of acetone from CO_2 in *Synechococcus elongatus* PCC 7942 under light and aerobic condition. *Plant Biotechnol J* **14**, 1768-1776 (2016).

15. Li, C. et al. Enhancing the light-driven production of D-lactate by engineering cyanobacterium using a combinational strategy. *Sci Rep* **5**, 9777 (2015).

16. Atsumi, S., Higashide, W. & Liao, J.C. Direct photosynthetic recycling of carbon dioxide to isobutyraldehyde. *Nat Biotechnol* **27**, 1177-1180 (2009).

17. Wang, Y. et al. Biosynthesis of platform chemical 3-hydroxypropionic acid (3-HP) directly from CO_2 in cyanobacterium *Synechocystis* sp. PCC 6803. *Metab Eng* **34**, 60-70 (2016).

18. Hirokawa, Y., Maki, Y., Tatsuke, T. & Hanai, T. Cyanobacterial production of 1,3-propanediol directly from carbon dioxide using a synthetic metabolic pathway. *Metab Eng* **34**, 97-103 (2016).

19. Shiho, M. et al. Business evaluation of a green microalgae *Botryococcus Braunii* oil production system. *Procedia Environmental Sciences* **15**, 90-109 (2012).

20. Shaw, A.J. et al. Metabolic engineering of microbial competitive advantage for industrial fermentation processes. *Science* **353**, 583-586 (2016).

21. Xiu, Z.L. & Zeng, A.P. Present state and perspective of downstream processing of biologically produced 1,3-propanediol and 2,3-butanediol. *Appl Microbiol Biotechnol* **78**, 917-926 (2008).
